# Insecticidal and Repellent Activity of Essential Oils from Seven Different Plant Species against *Tribolium castaneum* (Coleoptera: Tenebrionidae)

**DOI:** 10.3390/insects15100755

**Published:** 2024-09-29

**Authors:** Misha Khalil, Mishal Khizar, Dalal Suleiman Alshaya, Asifa Hameed, Noor Muhammad, Muhammad Binyameen, Muhammad Azeem, Mussurat Hussain, Qaisar Abbas, Kotb A. Attia, Tawaf Ali Shah

**Affiliations:** 1 Department of Entomology, Faculty of Agricultural Sciences & Technology, Bahauddin Zakariya University, Multan 60000, Pakistan; mishakhalil09@gmail.com (M.K.); mishalumair33@gmail.com (M.K.); mbinyameen@bzu.edu.pk (M.B.); 2 Entomological Research Sub-Station, Multan 60000, Pakistan; mussurratent@gmail.com (M.H.); abbas603@gmail.com (Q.A.); 3 Department of Biology, College of Science, Princes Nourah bint Abdulrahman University, P.O. Box 84428, Riyadh 11671, Saudi Arabia; 4 Department of Entomology, Mango Research Institute, Multan 60000, Pakistan; 5 Central Cotton Research Institute, Multan 60000, Pakistan; noor.1272@yahoo.com; 6 Department of Chemistry, COMSATS University Islamabad, Abottabad Campus, Abottabad 22060, Pakistan; muhazeem@cuiatd.edu.pk; 7 Centre of Excellence in Biotechnology Research, King Saud University, P.O. Box 2455, Riyadh 1145, Saudi Arabia; kattia1.c@ksu.edu.sa; 8 College of Agriculture Engineering and Food Science, Shandong University of Technology, Zibo 255000, China; tawafbiotech@yahoo.com

**Keywords:** red flour beetle, *Chinopodium ambrosiodes*, *Pinus roxburghii*, *Zanthoxylum armatum*, *Lepidium sativum*, *Azadiracta indica*, *Baccharis teindalensis*, *Origanum majorana*, repellent potential, fumigant toxicity

## Abstract

**Simple Summary:**

The red flour beetle is the most destructive pest of stored grain commodities, including flour, food grains, biscuits, pasta, nuts, cereals, and beans. Warehouse owners mostly use fumigants to control its infestation; however, these fumigants are toxic. The Indo–Pakistan region is rich in certain plant species that have repellent or deterrent effects on insects, and these plants are safer for humans. Among these plants are Mexican tea, long leaf Indian pine, rattan pepper, garden cress, neem, *Baccharis teindalensis* (a Columbian native herb also found in Pakistan), and marjoram. Hence, in the present study, we determined the toxic effect of these essential oil extracts against red flour beetle. The toxicity and repellent activities of these plant oils were determined through four-arm olfactometer bioassays. Mortality was recorded 1 day, 2 days, and 3 days after oil extract exposure. Data were analyzed using statistical software. Means were compared through LSD at the 5% level of significance. Overall, Mexican tea and Indian native palm oil were effective repellents. This study concludes that Mexican tea and Indian native palm oil can be used as alternative repellents against red flour beetle infestation.

**Abstract:**

*Tribolium castaneum* (Herbst) (Coleoptera: Tenebrionidae) is the most destructive pest of stored grain commodities. To control the attack of this insect pest, it is important to develop non-hazardous alternatives to replace fumigants. This study examined the fumigant toxicity and repellent activity of seven essential oils (*Chinopodium ambrosiodes, Pinus roxburghii, Zanthoxylum armatum*, *Lepidium sativum, Azadirachta indica, Baccharis teindalensis*, and *Origanum majorana*) against adult *T. castaneum* under controlled laboratory conditions. The fumigant toxicity and repellent activities of essential oils were tested using five different doses (62.5, 125, 250, 500, and 1000 µg) in vapour-phase fumigation and four-arm olfactometer bioassays, respectively. In vapor-phase fumigation bioassays, mortality data were recorded after 24, 48, and 72 h. The results showed that *C. ambrosiodes* and *P. roxburghii* essential oils are potential fumigants against adult *T. castaneum*. In repellency bioassays, a one-week-old adult population of *T. castaneum* was used to test the repellency potential of the essential oils. The results indicated that *C. ambrosiodes* and *P. roxburghii* had significant repellency potential against *T. castaneum*. Overall, we conclude that these essential oils have strong repellent and fumigant properties and can be used as potential repellent compounds to deter the insects.

## 1. Introduction

The red flour beetle, *T. castaneum* (Herbst) (Coleoptera: Tenebrionidae), is a cosmopolitan insect pest and can be found in flour mills [1], store grain warehouses [2], and grocery shops [3]. *Tribolium castaneum* is a secondary pest of stored grains because it prefers feeding on damaged and broken grains or grains already infested by other pests [4]. Both adult and larva of *T. castaneum* feed on broken grains and directly affect the quality and quantity of stored grains and their products [5]. For example, infested grain quality is highly reduced in terms of nutritional and aesthetic value, making products unmarketable and unfit for human consumption [6]. *Tribolium castaneum* may cause huge quantitative losses in the form of reduced grain weight [7,8].

The protection of stored grain and its commodities from the losses of stored insect pests is very important to ensure global food security [9]. Many chemical insecticides used to control stored grain insect pests are now restricted due to their poisonous effects on non-target species, including humans and the environment [10,11]. Moreover, insecticide resistance against commercial insecticides caused the over-use of insecticides, resulting in toxic effects to the environment [12]. Methyl bromide and phosphine are the most commonly used fumigants for stored grain insect pests, but their use is restricted due to serious health risks to humans [13,14,15]. The continuous use of toxic insecticides against red flour beetle may also lead to pesticide resistance against insect pests. The continuous use of phosphine has also showed negative effects on the growth and development of stored grain insect pests [16].

Alternatively, there is a growing interest in research concerning the use of plant-derived compounds. Plant products are biodegradable and generally less toxic to humans, non-target species, and the environment [6,17]. Volatiles emitted from plants or plant-derived materials, such as plant essential oils, have shown strong insecticidal, fumigation, and repellent properties against stored grain pests [18] and negatively affect their fitness. For example, essential oils from *Mentha arvensis*, *M. piperate*, and *M. spicata* reduce the adult emergence rate and egg-laying capacity of the stored cowpea pest *Callosobruchus maculates* (F.) (Coleoptera: Bruchidae) [19].

Plant-derived essential oils, such as being not at all or the least harmful, unique in action, environmentally friendly, and safe to apply, would make them the preferred option to be used against stored grain insect pests. The expected fumigant mode of action of these botanical oils might be through penetration into the air spaces of grains, where they act as a repellent or induce the mortality of *T. castaneum* through its direct contact with these essential oils. The present study evaluated the repellent response and fumigant potential of seven plant-derived essential oils against adult *T. castaneum*. The repellent potential and toxicity of seven essential oils, viz., *Chinopodium ambrosiodes, Pinus roxburghii*, *Zanthoxylum armatum*, *Lepidium sativum*, *Azadirachta indica*, *Baccharis teindalensis*, and *Origanum majorana*, were assessed through olfactometer and vapor-phase toxicity bioassays, respectively.

## 2. Materials and Methods

### 2.1. Insect Rearing

The experiment was conducted at Bhauddin Zakariya University, Multan. Mass culture of the red flour beetle, *T. castaneum*, was established in the laboratory without any exposure to chemical insecticides. The culture was maintained in plastic jars (1.5 L) containing wheat flour mixed with yeast (8:2) on weight basis. The plastic jars were covered with muslin cloth for ventilation. The culture was kept under controlled conditions (27 ± 2 °C; 65 ± 5% RH) in the dark. Jars were checked every 7th day, and newly emerged adults were separated from culture media using 60 mm mesh and put into new jars. Adult beetles aged 1–7 days old were used for toxicity and repellency bioassays.

### 2.2. Collection of Plant Materials and Extraction of Essential Oils

Fresh aerial parts of the plants (*C. ambrosiodes*, *P. roxburghii*, *Z. armatum*, *L. sativum*, *A. indica*, *B. teindalensis* and *O. majorana*) were collected from Abbottabad, Pakistan, during July–August 2016. Collected plants were identified by a taxonomist at the Department of Environmental Science, COMSATS University Islamabad, Abbottabad campus, Abbottabad, Pakistan. Fresh plant parts were kept in a freezer at −20 °C for at least 24 h prior to extraction. Essential oils of collected plants were extracted by using a stainless-steel distillation apparatus following Rao and Pandey [20] with some modifications.

The aerial parts of each collected plant sample were crushed into small pieces with a knife and weighed. About 1500 g of each sample was then placed in a sieve compartment of a vessel with 2 L of distilled water. An electric hot plate was used to heat the vessel. Due to heating pressure, the condensed water vapors moved through the vessel and plant sample present on the sieved compartment. The packed plant sample released volatile compounds, which were then cooled into a distillate by a condenser. The distillate was collected for 4 h and was subjected to liquid extraction using n-hexane. Essential oils extracted at different time intervals per plant species were pooled and dried over anhydrous magnesium sulfate to remove water. Solvent was evaporated by rotary evaporator to reduced pressure at 25 °C. Each essential oil sample was weighed to determine percentage yield. Finally, extracted essential oils were stored at −20 °C in a glass vial until analysis and used for fumigation and repellent bioassays.

### 2.3. Fumigation Bioassay

The toxicity of essential oils on *T. castaneum* adults was tested by vapor-phase toxicity bioassay in 15 mL glass vials [21]. Seven different essential oils (*C. ambrosiodes*, *P. roxburghii*, *Z. armatum*, *L. sativum*, *A. indica*, *B. teindalensis* and *O. majorana*) were used in a fumigation bioassay. A strip (1 cm wide and 5 cm long) of Whatman No. 1 filter paper was taken, and a 10 µL aliquot of different essential oil concentrations (100 µg/mL, 50 µg/mL, 25 µg/mL, 12.5 µg/mL, 6.12 µg/mL) was prepared separately and kept at room temperature for 2–3 min to evaporate the solvent (ethanol). For each replicate, a filter strip was suspended in the glass vial, and tube cap was screwed tightly after adding 10 adult beetles and 1 g of crushed grains into each tube. An ethanol filter paper strip was used as a negative control. Each treatment and negative control was replicated five times. Mortality was checked after 24, 48, and 72 h following exposure. Adults were considered dead if appendages did not move when prodded with a fine entomological pin.

### 2.4. Repellency Bioassays

**Figure 1:**
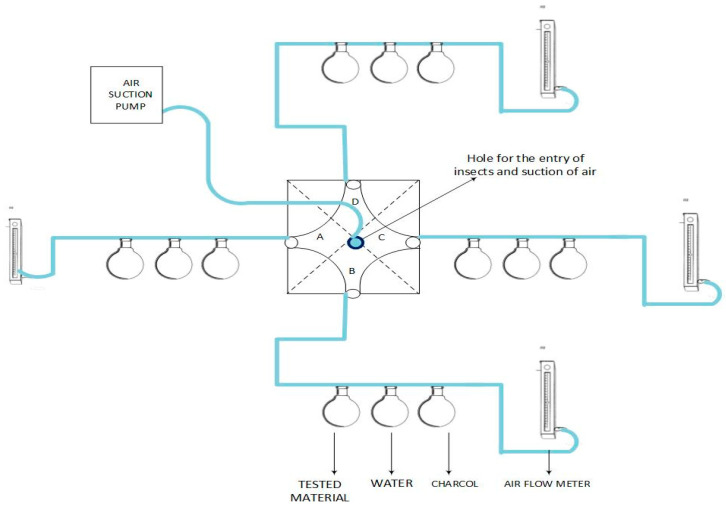
Schematic drawing of the four-arm olfactometer used in the bioassays. Here A, B, C, and D are four opening of four armed olfactometer.

A four-arm olfactometer was used to evaluate the response of *T. castaneum* adults to different treatments: crushed grains, crushed grains + essential oils, ethanol + crushed grains, and empty as a control. The glass olfactometer had an upper and lower layer with dimensions of 12 × 12 × 1.2 cm and 10.2 × 10.2 × 0.6 cm, respectively. These two glass layers consisted of four openings along four regions (Figure 1). A small outlet (4 mm diameter) was present in the center upper glass plate for a *T. castaneum adult* to enter. Each arm of the olfactometer was made of socket glasses that were connected with the three glass vials (50 mL). Each glass vial contained either the treatment, a charcoal purification filter or water treatment. Air pressure was controlled using an air flow meter (4 Pa). A battery having dual-pump system was used to regulate within olfactometer.

The air flow was passed through the air entrainment system (KNF Neuberger, Freiburg Berisgau, Germany) through Teflon tubing measuring 3.2 × 1.5 (Camlab Ltd., Cambridge, UK). Adult beetles were singly placed in the center of olfactometer, the position of beetles was observed visually, and data were recorded for five minutes using a stopwatch. In each bioassay, twenty replicates (individual beetles) were used to test the response of *T. castaneum* to treatments. The number of entries per treatment and time spent in each treatment were recorded. An initial bioassay was performed to check beetle responsiveness, where two olfactometer arms contained 2 g of crushed grain and the other two arms contained 2 g whole grain.

This was followed by the repellency bioassay. One arm contained filter paper impregnated with different concentrations of essential oils (62.5 µg, 125 µg, 250 µg, 500 µg, and 1000 µg) and crushed grain. The other three arms contained 2 positive controls: (i) 2 g of crushed grains; (ii) crushed grains + strip of filter paper impregnated with ethanol and one negative control (empty arm). In all bioassays, 10 µL of different concentrations of essential oils was applied on filter paper. The olfactometer experiment involved uniform exposure under a fluorescent light.

### 2.5. Statistical Analysis

Statistics 8.1 software was used to analyze experimental data. Time spent by *T. castaneum* in the four different treatments (arms) of the olfactometer was evaluated using one-way ANOVA. In fumigation bioassays, the mean mortality rate of *T. castaneum* was evaluated using one-way ANOVA, and a comparison was made through LSD test at 5% level of significance.

## 3. Results

### 3.1. Repellent Effects of C. ambrosiodes against T. castaneum

**Figure 2:**
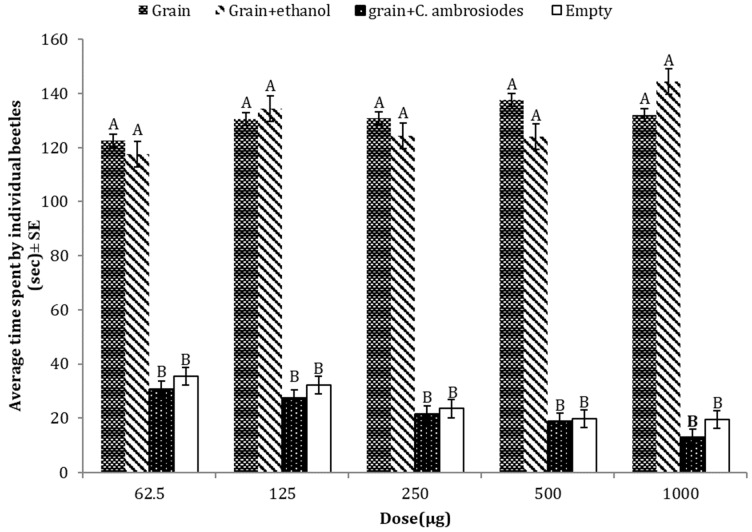
Average time spent by *T. castaneum* in response to different doses of *C. ambrosiodes* essential oil as compared to three controls (Grain, Grain + solvent, Empty). Red flour beetle spent less time in grain + *C. ambrosides* compared to other treatments in all doses 62.5 µg, 125 µg, 250 µg, 500 µg, and 1000 µg. Here the letters (A,B) represents the ranks obtained by comparing means among treatments through Tukey HSD at 5% level of significance.

The results of four-arm olfactometer bioassays showed that the tested oils inhibited the attraction of the *T. castaneum* towards food material. Adult *T. castaneum* spent less time in arms either emitting volatile compounds of *C. ambrosiodes* essential oil with grain (food bait) or in the empty arm (negative control), as compared to the two other treatments (grains or grains + ethanol). The results of one-way ANOVA showed that oil maintained potential significant repellency at different doses i.e., 62.5 µg (*p* = 0.000, F = 8.92, DF = 3), 125 µg (*p* = 0.0000, F = 10.1, DF = 3), 250 µg (*p* = 0.0000, F = 10.5, DF = 3; 500 µg: *p* = 0.0000, F = 15.0 DF = 3; and 1000 µg: *p* = 0.0000, F = 16.6, DF = 3) (Figure 2).

### 3.2. Repellent Effects of P. roxburghii against T. castaneum

**Figure 3:**
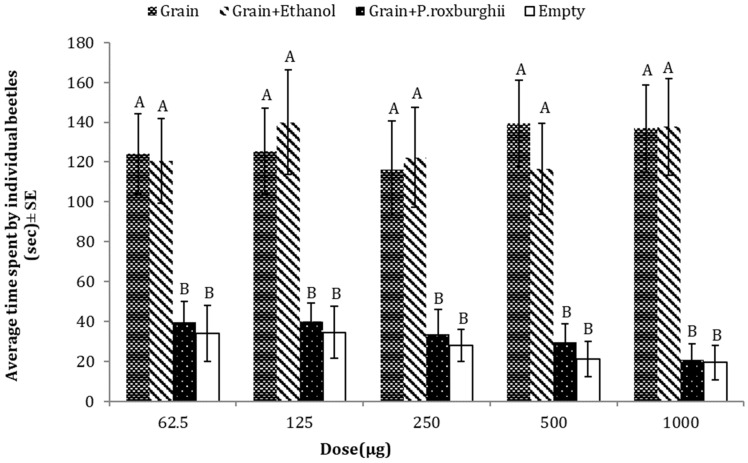
Average time spent by *T. castaneum* in response to different doses of *P. roxburghii* essential oil as compared to three controls (grain, grain + solvent, empty). Overall, the red flour beetle spent significantly less time in grain + *P. roxburgii.* Here the letters (A,B) represents the ranks obtained by comparing means among treatments through Tukey HSD at 5% level of significance.

*P. roxburghii* oils inhibited the attraction of *T. castaneum* towards food material. Adult *T. castaneum* spent less time in arms either emitting volatile compounds of *P. roxburghii* essential oil with grain (food bait) or in the empty arm (negative control), as compared to the two other treatments (grains or grains + solvent (ethanol)). The result of one-way ANOVA showed that oil had a great repellent potential at different doses of essential oil (62.5 µg: *p* = 0.0002, F = 7.65, DF = 3; 125 µg: *p* = 0.0001, F = 8.03, DF = 3; 250 µg: *p* = 0.0002, F = 7.28, DF = 3; 500 µg: *p* = 0.0000, F = 12.5, DF = 3; 1000 µg: *p* = 0.0000, F = 14.9, DF = 3) (Figure 3).

### 3.3. Repellent Effects of Z. armatum against T. castaneum

**Figure 4:**
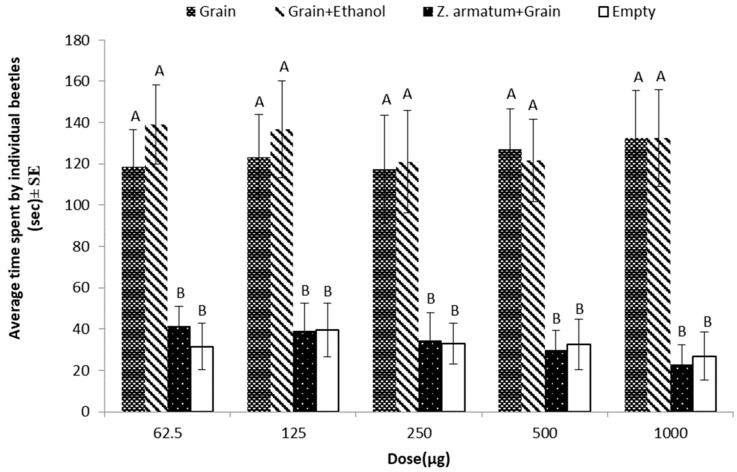
Average time spent by *T. castaneum* in response to different doses of *Z. armatum* seed essential oil as compared to three controls (grain, grain + solvent, empty). Here the letters (A,B) represents the ranks obtained by comparing means among treatments through Tukey HSD at 5% level of significance.

*Z. armatum* oil inhibited the attraction of *T. castaneum* towards the food material. Adult *T. castaneum* spent less time in the arms either emitting volatile compounds of *Z. armatum* seed essential oil with grains (food bait) or in the empty arm (negative control), as compared to the two other treatments (grains or grains + solvent (ethanol)). The result of one-way ANOVA showed that *Z. armatum* seed oil has great repellency potential at all tested doses towards adult beetles of *T. castaneum* (e.g., 62.5 µg: *p* = 0.0000, F = 11.8, DF = 3; 125 µg: *p* = 0.0002, F = 7.60, DF = 3; 250 µg: *p* = 0.0008, F = 6.18, DF = 3; 500 µg: *p* = 0.000, F = 10.6, DF = 3; 1000 µg: *p* = 0.0000, F = 11.6, DF = 3) (Figure 4).

### 3.4. Repellent Effects of L. sativum against T. castaneum

**Figure 5:**
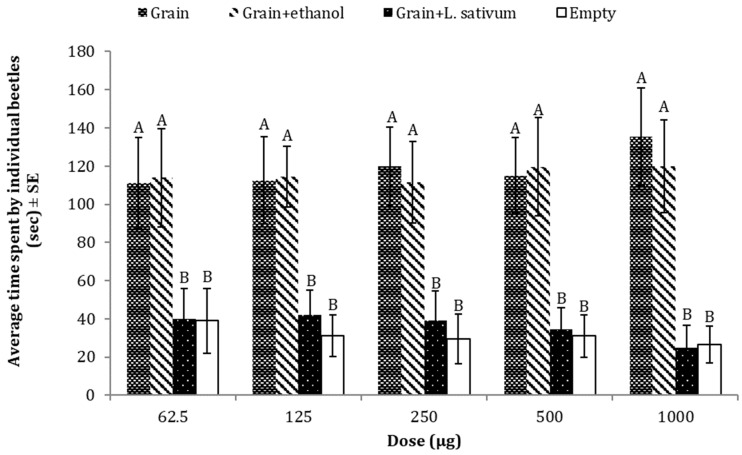
Average time spent by *T. castaneum* in response to different doses of *L. sativum* seed essential oil as compared to three controls (grain, grain + solvent, empty). Here, the letters (A,B) represent the ranks obtained by comparing means among treatments through Tukey HSD at 5% level of significance.

*L. sativum* oil inhibited the attraction of *T. castaneum* towards the food material. Adults of *T. castaneum* spent significantly less time in the arm of the olfactometer, emitting either volatile compounds of *L. sativum* essential oil in addition to the grains (food bait) or in the empty arm (negative control), as compared to the two other treatments (grains or grains + solvent (ethanol)). The result of one-way ANOVA showed that oil maintained potential repellency at different doses of essential oil (e.g., 625 µg: *p* = 0.0003, F = 8.92, DF = 3; 125 µg: *p* = 0.0002, F = 7.59, DF = 3; 250 µg: *p* = 0.0003, F = 6.96, DF = 3; 500 µg: *p* = 0.0002, F = 7.23, DF = 3; 1000 µg *p* = 0.0000, F = 9.45, DF = 3) (Figure 5).

### 3.5. Repellent Effects of A. indica against T. castaneum

**Figure 6:**
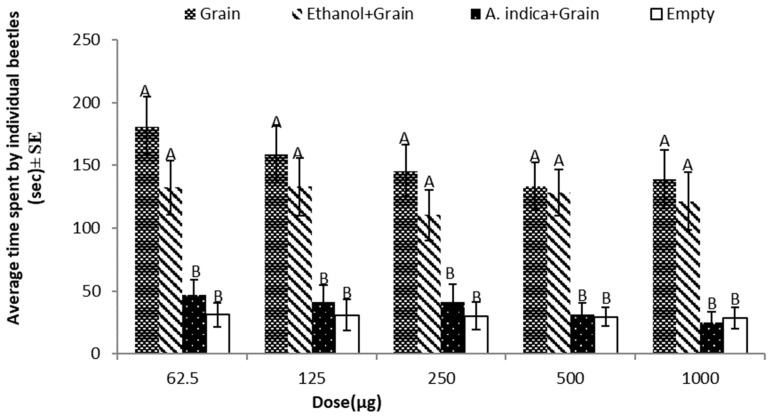
Average time spent by *T. castaneum* in response to different doses of *A. indica* essential oil as compared to three controls (grain, grain + solvent, empty). Here, the letters (A,B) represent the ranks obtained by comparing means in among treatments through Tukey HSD at 5% level of significance.

*A. indica* oils inhibited the attraction of *T. castaneum* towards the food material. Adults of *T. castaneum* spent significantly less time in the arm of the olfactometer, emitting either volatile compounds of *A. indica* essential oil in addition to the grains (food bait) or in the empty arm (negative control), as compared to the two other treatments (grains or grains + solvent (ethanol)). The result of one-way ANOVA showed that oil maintained potential repellency at different doses of essential oil (62.5 µg: *p* = 0.000, F = 13.1, DF = 3; 125 µg: *p* = 0.0000, F = 10.6, DF = 3; 250 µg: *p* = 0.0000, F = 9.21, DF = 3; 500 µg: *p* = 0.0000, F = 14.7 D, F = 3; 1000 µg: *p* = 0.0000, F = 11.6, DF = 3) (Figure 6).

### 3.6. Repellent Effects of B. teindalensis against T. castaneum

**Figure 7:**
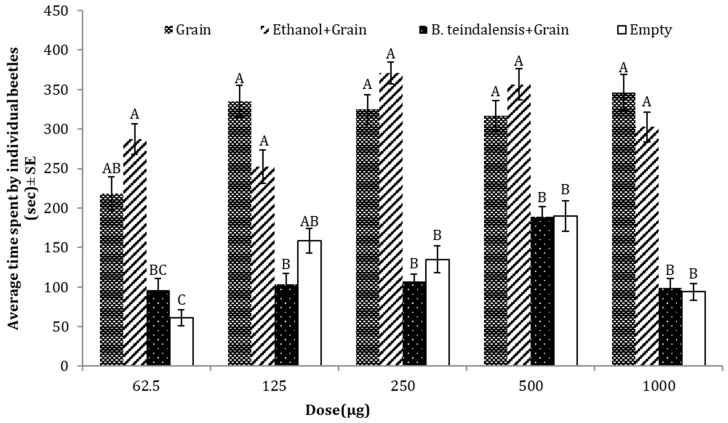
Average time spent by *T. castaneum* in response to different doses of *B. teindalensis* essential oil as compared to three controls (grain, grain + solvent, empty). Here, the letters (A,B,C) represent the ranks obtained by comparing means in among treatments through Tukey HSD at 5% level of significance.

The result of four-arm olfactometer bioassays showed that the tested oil inhibited the attraction of *T. castaneum* towards the food material. Adults of *T. castaneum* spent significantly less time in the arm of the olfactometer, emitting either volatile compounds of *B. teindalensis* essential oil in addition to the grains (food bait) or in the empty arm (negative control), as compared to the two other treatments (grains or grains + solvent (ethanol)). The result of one-way ANOVA showed that oil maintained strong repellency at different doses of essential oil (62.5 µg: *p* = 0.0000, F = 8.79, DF = 3; 125 µg: *p* = 0.0000, F = 14.4, DF = 3; 250 µg: *p* = 0.0001, F = 7.87, DF = 3; 500 µg: *p* = 0.0000, F = 8.78, DF = 3; 1000 µg: *p* = 0.0000, F = 11.1, DF = 3) (Figure 7).

### 3.7. Repellent Effects of O. majorana against T. castaneum

**Figure 8:**
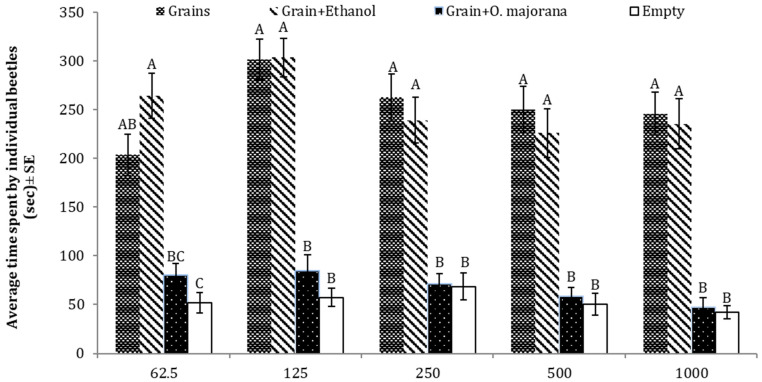
Average time spent by *T. castaneum* in response to different doses of *O. majorana* seed essential oil as compared to three controls (grain, grain + solvent, empty). Here, the letters (A,B,C) represent the ranks obtained by comparing means iamong treatments through Tukey HSD at 5% level of significance.

The results of the behavior bioassay, conducted in the four-arm olfactometer, showed that the tested oils inhibited the attraction of *T. castaneum* towards the food material. Adults of *T. castaneum* spent significantly less time in the arm of the olfactometer, emitting either volatile compounds of *O. majorana* oil in addition to the grains (food bait) or the empty arm (negative arm), as compared to the two other treatments (grains or grains + solvent (ethanol)). The result of one-way ANOVA showed that essential oil was a great repellent potential towards adult beetles of *T. castaneum* at different doses of essential oil (62.5 µg: *p* = 0.0000, F = 8.79, DF = 3; 125 µg: *p* = 0.0000, F = 14.4, DF = 3; 250 µg: *p* = 0.0001, F = 7.87, DF = 3; 500 µg *p* = 0.0000, F = 8.78, DF = 3; 1000 µg *p* = 0.0000, F = 11.1, DF = 3) (Figure 8).

### 3.8. Insecticidal Effects of Essential Oils in T. castaneum after 24 h

**Figure 9:**
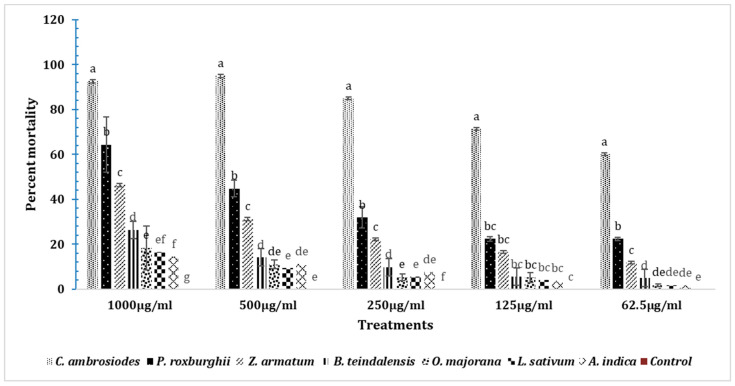
Fumigation toxicity at different doses of essential oils after 24 h against *T. castaneum* adults. The one-way ANOVA analysis was performed separately for each dose through Statistix 8.1. Here, the letters a–g represent ranks obtained through comparing means through Tukey HSD at 5% level of significance.

The one-way ANOVA analysis of mortality caused by fumigants at 1000 µg/mL after 24 h showed significant differences (DF = 7; F = 2289; *p* < 0.01). The highest mortality of adult *T. casteneum* was caused by *C. ambrosiodes* essential oil at all tested doses (1000 µg, 500 µg, 250 µg, 125 µg and 62.5 µg) after 24 h, as compared to the respective doses of all other essential oils tested (Figure 9). The mortality pattern showed that at a 1000 µg dose, *C. ambrosiodes* caused the highest mortality (92.6%), followed by *P. roxburghii* (64.4%), Z. *armatum* (46.4%), *B. teindalensis* (26.4%), *O. majorana* (18.4%), *A. indica* (16.6%), and *L. sativum* (14.4%). Likewise, at a 500 µg dose, the highest mortality was observed in the adults exposed to *C. ambrosiodes* (95%), followed by *P. roxburghii* (44.8%) and *Z. armatum* (31.2%), *B. teindalensis* (14.2%), *O. majorana* (11.2%), *A. indica* (11%), and *L. sativum* (9.4%) (DF = 7; F = 1041; *p* = 0.00). Likewise, at a 250 µg dose, the highest mortality was observed in adults exposed to *C. ambrosiodes* (85%), followed by *P. roxburghii* (32%), *Z. armatum* (22.2%), *B. teindalensis* (9.8%), *O. majorana* (7.6%), *A. indica* (5.6%), and *L. sativum* (5.4%) (DF = 7; F = 1041; *p* = 0.00). Similarly, at a 125 µg dose, a significant difference in mortality was observed (DF = 7; F = 3.70; *p* = 0.004). At a 125 µg dose, the highest mortality was observed in adults exposed to *C. ambrosiodes* (71.4%), followed by *P. roxburghii* (22.4%) and *Z. armatum* (16.6%), *B. teindalensis* (5.6%), *O. majorana* (5.2%), *L. sativum* (4%), and *A. indica* (3.8%). Likewise, at a 62.5 µg dose, the highest mortality was observed in adults exposed to *C. ambrosiodes* (60.1%), followed by *P. roxburghii* (22.4%), *Z. armatum* (11.8%), *B. teindalensis* (5%), *O. majorana* (1.8%), *L. sativum* (1.8%), and *A. indica* (1.6%).

### 3.9. Insecticidal Effects of Essential Oils on T. castaneum after 48 h

**Figure 10:**
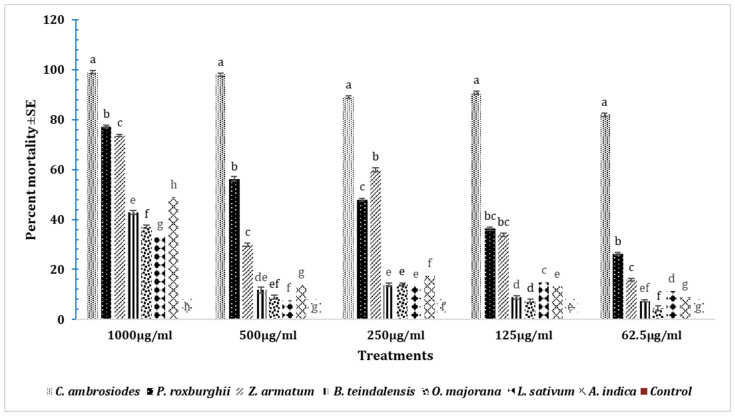
Fumigation toxicity at different doses of essential oils after 72 h against *T. castaneum* adult. The one-way ANOVA analysis was done separately for each dose through Statistix 8.1. Here, the letters a–h represent ranks obtained through comparing means through Tukey HSD at 5% level of significance. Insecticidal effects of essential oils in *T. castaneum* after 72 h.

The highest mortality of adult *T. castaneum* was caused by *C. ambrosiodes* essential oil at all tested doses (1000 µg, 500 µg, 250 µg, 125 µg and 62.5 µg) after 48 h, as compared to the respective doses of all other essential oils tested (Figure 10). The mortality pattern showed that at a 1000 µg dose, *C. ambrosiodes* caused the highest mortality (99%), followed by *P. roxburghii* (77.2%), *Z. armatum* (73.6%), *A. indica* (49%), *B. teindalensis* (42.8%), *O. majorana* (37%), *L. sativum* (33%), and control (0%) (DF = 7; F = 2290; *p* = 0.00) (Figure 10). Likewise, at a 500 µg dose, the highest mortality was observed in adults exposed to *C. ambrosiodes* (98%), followed by *P. roxburghii* (56.2%), *Z. armatum* (29.8%), *A. indica* (29.8%), *B. teindalensis* (11.8%), *O. majorana* (9%), *L. sativum* (7.6%), and control (0%) (DF = 7; F = 2311; *p* = 0.00) (Figure 10). Likewise, at a 250 µg dose, the highest mortality was observed in adults exposed to *C. ambrosiodes* (89%), followed by *P. roxburghii* (48%) *Z. armatum* (60%), *B. teindalensis* (13.8%), *O. majorana* (9%), *L. sativum* (7.6%), and control (0.0%) (DF = 7; F = 3272; *p* = 0.00) (Figure 10). Likewise, at a 125 µg dose, the highest mortality was observed in *C. ambrosiodes* (90.8%), followed by *P. roxburghii* (36.4%), *Z. armatum* (34%), *L. sativum* (14.8%), *A. indica* (13.2%), *B. teindalensis* (7.4), *O. majorana* (7.4%), and control (0.0%) (DF = 7; F = 2577; *p* = 0.00). The three oils *(B. teindalensis, O. majorana*, *L. sativum*) caused the lowest mortality in all doses after 48 h of adult beetle exposure. Likewise, at a 62.5 µg dose, the highest mortality was observed in adults exposed to *C. ambrosiodes* (82%), followed by *P. roxburghii* (26.2%), *Z. armatum* (15.8%), *L. sativum* (11.2%), *A. indica* (9%), *B. teindalensis* (7.4%), *O. majorana* (4.4%), and control (DF = 7; F = 1421; *p* = 0.00) (Figure 10).

**Figure 11:**
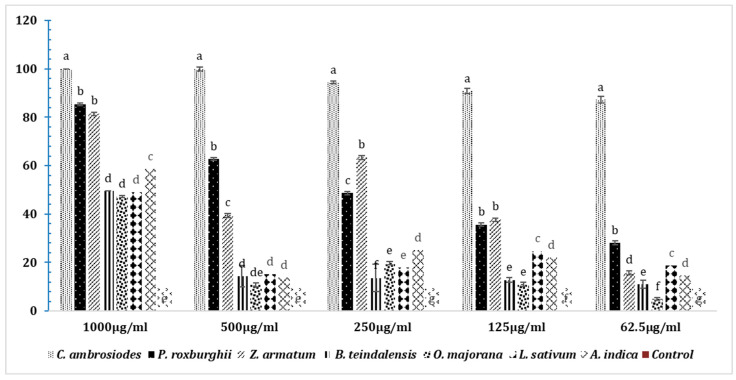
Fumigation toxicity at different doses of essential oils after 72 h against *T. castaneum* adults. The one-way ANOVA analysis was done separately for each dose through Statistix 8.1. Here, the letters a–f represent ranks obtained through comparing means through Tukey HSD at 5% level of significance.

The one-way ANOVA analysis of mortality caused by fumigants at 1000 µg/mL after 24 h showed a significant difference (DF = 7; 997; *p* < 0.00). The highest mortality of adult *T. castaneum* was caused by *C. ambrosiodes* essential oil at all tested doses (1000 µg, 500 µg, 250 µg, 125 µg and 62.5 µg) after 72 h, as compared to the respective doses of all other essential oils tested (Figure 11). At a 1000 µg dose, *C. ambrosiodes* caused the highest mortality (100%), followed by *P. roxburghii* (85.4%) *Z. armatum* (81.6%), *A. indica* (58.6%), *B. teindalensis* (49.6%), *L. sativum* (49%), *O. majorana* (47.2%), and control (Figure 11). Likewise, at a 500 µg dose, the highest mortality was observed in adults exposed to *C. ambrosiodes* (100%), followed by *P. roxburghii* (62.8%), *Z. armatum* (39.6%), *L. sativum* (15.2%), *B. teindalensis* (14.4%), *A. indica* (13.8%), *O. majorana* (10.8%), and control (0.0%) (DF = 7; F = 164; *p* = 0.00) (Figure 11). Likewise, at a 250 µg dose, the highest mortality was observed in adults exposed to *C. ambrosiodes* (94.4%), followed by *P. roxburghii* (48.6%) *Z. armatum* (63.6%), *A. indica*, *B. teindalensis* (13.6%), *O. majorana* (19.8%), *L. sativum* (17.8%), and control (0.0) (DF = 7; F = 3687; *p* = 0.00) (Figure 11). Likewise, at a 125 µg dose, the highest mortality was observed in *C. ambrosiodes* (90.8%), followed by *P. roxburghii* (35.6%), *Z. armatum* (37.8%), *A. indica* (12%), *B. teindalensis* (11%), *O. majorana* (7.4%), *L. sativum* (14.8%), and control (0.0%) (DF = 7; F = 2446; *p* = 0.00). The three oils (*B. teindalensis*, *O. majorana*, *L. sativum*) caused the lowest mortality in all doses after 72 h of adult beetle exposure. Likewise, at a 62.5 µg dose, the highest mortality was observed in adults exposed to *C. ambrosiodes,* followed by *P. roxburghii Z. armatum*, *A. indica*, *B. teindalensis*, *O. majorana*, *L. sativum*, and control (DF7; F = 1951; *p* = 0.00) (Figure 11).

## 4. Discussion

Plant-based botanical insecticides are environmentally friendly, safer for humans, less expensive, and biodegradable. Plant products such as essential oils are considered one of the most important natural chemicals to control insect pests of stored grains. It has been shown that essential oils exhibit insecticidal toxicity, anti-feeding, and repellence or deterrence against stored grain insect pests [22,23]. The toxic effects of using certain botanical essential oils against stored grain insect pests via fumigation have been reported by many scientists [24,25,26]. In our study, seven different essential oils exhibited good repellent activity and fumigant toxicity against adults of *T. castaneum* via four-arm olfactometer bioassays and fumigation assays. The results of our study are correlated with previous investigations. For example, Lee, Annis, and Choi [26] showed that *Eu. nicholi*, *Eu. blakeli*, *Eu. codonocarpa*, *Melleuca fulgens*, *Callistemon sieberi*, and *M. armillaris* essential oils were toxic against three stored grain insect species: *T. castaneum*, *S. oryzae*, and *R. dominica*. *O. vulgare* essential oil and its components showed potential as fumigants and repellents against adult *T. castaneum* [25].

Our study found that *C. ambrosiodes* essential oil repelled and killed *T. castaneum*. This effect may be due to the presence of chemical compounds in the oil, such as α-terpinene (30.3%), p-cymene (13.5%), ascaridole (30.6%), and isoascaridole (23.6%). Several studies have characterized the chemical composition, fumigation toxicity, and repellent potential of compounds found in *C. ambrosiodes* essential oil. For example, Kasali et al. [27] reported that *C. ambrosiodes* essential oil contained major components, such as α-terpinene (55.55%), p-cymene (16.71%), β-pinene (0.29%), limonene (1.09%), sabinene (1.50%), γ-terpinene (0.97%), phytol (0.38%), 1,4-epoxy-p-menth-2-ene (17.72%), (E)-β-ocimene (0.27%), and 1,2,3,4-diepoxy-p-menthane (0.14%). Chu et al. [28] reported that *C. ambrosiodes* essential oil contains five active components: *α*-terpinene, *ρ*-cymene, (*Z*)-ascaridole, isoascaridole, and 2-carene. They also reported the fumigant and contact toxicity of these components against the stored grain pest, *S. zeamais*. Previously, *C. ambrosioides* (powder and essential oil) has been used against six stored grain species: *C. maculates*, *P. truncates*, *C. chinensis*, *S. granarius*, *Acanthoscelides obtectus*, and *S. zeamais.* They caused high mortality [29].

*P. roxburghii* also showed great repellence and fumigant toxicity against adult *T. castaneum*. The results of our study are consistent with previous investigations. Three essential oils, *M. piperita*, *P. roxburghii*, and *Rosa* spp., showed strong fumigant toxicity against the stored grain insect pests: *T. castaneum*, *R. dominica*, and *S. oryzae*. The results showed that *P. roxburghii* was a potential fumigant against *R. dominica* and *S. oryzae,* with maximum percentage mortalities of 70% and 80%, respectively [30]. Similarly, another study also showed that essential oils of *A. sativum*, *Artimisia annua*, *Callistemon citrinus*, *C. botrys*, *Cinnamomum zeylanicum*, *Citrus reticulate*, *Cuminum cyminum*, *Foeniculum vulgare*, *Murraya koenigii*, *P. roxburghii*, *Piper nigram,* and *P. roxburghii* showed high insecticidal activity against *R. dominica*, *T. castaneum*, *S. oryzae*, and *C. chinensis* [31].

Similarly, *Z. armatum* also showed strong fumigant and repellent activity against adult *T. castaneum*. Further, some mosquito studies showed *Z. armatum* and its chemical components had higher larvicidal activity against *Culex quinquefasciatus* compared to *Aedes aegypti* and *Anopheles stephensi* [32]. Another report showed high toxicity of essential oil derived from *Z. armatum* and its components against *T. castaneum* and *Laisoderma serricorne*. Fumigation and contact toxicity were determined for these two stored grain insect pests. Both the fumigation and contact bioassays for *Z. armatum* essential oil showed high toxicity against *T. castaneum* and *L. serricorne* [33].

In our study, the remaining four essential oils (*A. indica*, *L. sativum*, *B. teindalensis*, and *O. majorana*) showed low mortality percentages in fumigant toxicity bioassays but were still moderately repellent toward adult *T. castaneum*. Previously, the repellent and fumigation potentials of different essential oils extracted from some indigenous plants such as *A. indica*, *D. stramonium*, and *Melia azedarach* were reported against three insect pests species: *R. dominica*, *T. castaneum*, and *T*. *granarium*. Two pest species (*T. castaneum* and *R. dominica*) were repelled by *A. indica* in experiments [34].

Previously, the repellent activity and fumigant toxicity of *L. sativum* to the 5th instar larvae of *T. granarium* showed strong larvicidal activity at different tested concentrations, while the repellency results showed that *L. sativum* essential oil was not repellent [35]. This indicates that an essential oil may have larvicidal activity but does not repel. Our results also showed varying activities in fumigation bioassays or the repellence bioassays.

Likewise, the repellent potential and fumigant toxicity of *O. majorana* essential oil extracted from leaves and flowers were tested against adult *T. castaneum* and larval *Plodia interpunctella*. The results showed that *O. majorana* essential oil from leaves caused more insecticidal activity towards adult *T. castaneum,* while the essential oil from flowers of *O. majorana* showed more repellent potential towards *T. castaneum* [36]. This indicates that an essential oil extracted from different stages or different parts of a plant may have varying levels of fumigation and repellent potential towards the target pests. Results from Padin et al. [37,38] also support our results, where *B. trimera* showed low insecticidal and repellence potential against *T. castaneum* adults.

## 5. Conclusions

Plant-derived essential oils and their constituents have a fumigant and repellent potential against *T. castaneum*. Three essential oils, e.g., *C. ambrosiodes*, *P. roxburghii,* and *Z. armatum,* exhibited strong fumigant toxicity and repellency through vapor-phase toxicity and olfactometer bioassays against adult *T. castaneum*. The information obtained from this study will enhance our knowledge about the potential use of these essential oils to control other stored grain insect pests. Finally, taking into account the fumigation toxicity and repellence results of these essential oils, future semi-field studies should be conducted. A critical analysis of the potential economic implications of these essential oils is required, so we further recommend the development of commercial products to be used in stored grain insect pest management.

## Data Availability

All data related to this study are contained within the research paper.
